# Regulatory role of exosomes in colorectal cancer progression and potential as biomarkers

**DOI:** 10.20892/j.issn.2095-3941.2023.0119

**Published:** 2023-08-08

**Authors:** Juan Hui, Mingzhen Zhou, Guangzhou An, Hui Zhang, Yuanyuan Lu, Xin Wang, Xiaodi Zhao

**Affiliations:** 1 Department of Gastroenterology, Tangdu Hospital, Air Force Medical University, Xi’an 710038, China; 2 State Key Laboratory of Cancer Biology and National Clinical Research Center for Digestive Diseases, Xijing Hospital of Digestive Diseases, Air Force Medical University, Xi’an 710032, China; 3 Department of Radiation Protection Medicine, Ministry of Education Key Laboratory of Hazard Assessment and Control in Special Operational Environment, Faculty of Preventive Medicine, Air Force Medical University, Xi’an 710032, China; 4 Department of Traditional Chinese Medicine, Tangdu Hospital, Air Force Medical University, Xi’an 710038, China

**Keywords:** Colorectal cancer, exosome, immunoregulation, immunotherapy, metastasis

## Abstract

Colorectal cancer (CRC) remains an enormous challenge to human health worldwide. Unfortunately, the mechanism underlying CRC progression is not well understood. Mounting evidence has confirmed that exosomes play a vital role in CRC progression, which has attracted extensive attention among researchers. In addition to acting as messengers between CRC cells, exosomes also participate in the CRC immunomodulatory process and reshape immune function. As stable message carriers and liquid biopsy option under development, exosomes are promising biomarkers in the diagnosis or treatment of CRC. In this review we have described and analyzed the biogenesis and release of exosomes and current research on the role of exosomes in immune regulation and metastasis of CRC. Moreover, we have discussed candidate exosomal molecules as potential biomarkers to diagnose CRC, predict CRC progression, or determine CRC chemoresistance, and described the significance of exosomes in the immunotherapy of CRC. This review provides insight to further understand the role of exosomes in CRC progression and identify valuable biomarkers that facilitate the clinical management of CRC patients.

## Introduction

According to data from the National Cancer Center of China, there were approximately 4.064 million newly diagnosed cancer cases in 2016. Of these cancer cases, colorectal cancer (CRC) is the second most common type of cancer diagnosed and the fifth leading cause of cancer-related deaths^1^. Interestingly, several studies have shown an increasing incidence of CRC in young people < 50 years of age^2^. Changes in dietary habits and adverse lifestyle factors, such as cigarette smoking, sedentary behavior, and physical inactivity, are the main reasons for this phenomenon^3,4^. The atypical CRC clinical symptoms lead to a low detection rate. Patients are often diagnosed in an advanced stage, which has a high mortality rate. Efficient and accurate screening methods are necessary for the prevention and control of CRC. At present, the early diagnosis of CRC mainly depends on colonoscopy. Although colonoscopy is the gold standard for diagnosing CRC, colonoscopy is an invasive procedure that causes patient discomfort and stress^5,6^. Therefore, it is necessary to find an ideal new biomarker for the diagnosis of CRC.

Exosomes are secreted by most cells in the body and can be detected in a variety of body fluids, including blood, urine, saliva, and breast milk^7,8^. Due to exosome advantages, including good stability and ability to carry diverse bioactive molecules, exosomes participate in communication between cells and regulate the biological behavior of cells^9^. Exosomes participate in tumor proliferation and metastasis, and regulate the tumor microenvironment (TME)^10^. Therefore, exosomes have a significant role in tumor development. In addition, studies have shown that exosomes are important nanomaterials with good biocompatibility and long-term stability in the blood circulation, thus exosomes can be used as targeted drug carriers for cancer therapy^11^. Therefore, exosomes have great potential and value for the diagnosis, prognostication, and treatment of tumors. In this review we discuss the biological characteristics of exosomes, the role of exosomes in immune regulation and metastasis of CRC, and the clinical application value of exosomes.

## Exosomes

### Overview of extracellular vesicles (EVs)

EVs are a group of heterogeneous vesicles with membranous structures that are secreted by most cells in the body, including both healthy cells and cancer cells^12^. According to the origin, biogenesis, and release pathway, EVs can be divided into three main types: apoptotic EVs; ectosomes; and exosomes^13^. Apoptotic EVs, also known as apoptotic bodies and vesicles, are produced by cells during apoptosis and range from 100 nm-5 μm in diameter^14^. Ectosomes are generated by direct outward budding of the cell plasma membrane and range from 30 nm-10 μm in diameter^13^. Moreover, depending on the size, molecular composition, and the specific mechanisms underlying the release pathway, ectosomes can be divided into microvesicles, large oncosomes, small ectosomes, and arrestin domain-containing protein 1-mediated microvesicles (ARMMs). Studies have shown that both healthy cells and cancer cells secrete microvesicles, small ectosomes, and ARMMs, while large oncosomes are only secreted by tumor cells^13^. Exosomes are nanoscale particles with a diameter ranging from 30-150 nm and are produced by a relatively complex mechanism^15^.

### Structure of exosomes

Exosomes are generally spherical, cup-shaped, or saucer-shaped membrane-bound EVs^16^. The exosome membrane contains lipids, including cholesterol, sphingomyelin, ceramides, and glycolipids^17,18^. The exosome membrane also contains transmembrane proteins, such as tetraspanins, molecular chaperones, adhesion molecules, and major histocompatibility complex (MHC) molecules^17,18^. Studies have shown that tetraspanins (CD63, CD81, and CD9) are highly enriched in exosomes and have been used as exosome marker proteins for a long time^19,20^.

### Biogenesis of exosomes

Exosomes are currently the most widely-studied EV subsets. It has been reported that exosome biogenesis is a three-stage process. First, an early endosome is formed by invagination of the plasma membrane, which then matures through the acidification and material exchange to form a late endosome. The late endosome eventually forms multivesicular bodies (MVBs) containing intraluminal vesicles (ILVs). When MVBs fuse with the plasma membrane, ILVs are released into the extracellular space; the released ILVs are called exosomes^21,22^. The biogenesis of exosomes mainly includes the following two sorting mechanisms: the endosomal sorting complex for transport (ESCRT) pathway; and the ESCRT-independent pathway^23,24^.

### Cargo sorting and exosome components

Exosomes contain a variety of biomolecules, such as DNA, microRNA(miRNA), long non-coding RNA (lncRNA), circular RNA (circRNA), transfer RNA, proteins, amino acids, lipids, and metabolites^25^. In addition, numerous studies have elucidated the mechanisms affecting the sorting of molecules to exosomes. Specifically, Yokoi et al.^26^ reported that tetraspanins directly interact with cancer cell micronuclei, leading to genomic DNA loading into exosomes. RNA-binding proteins promote the loading of miRNAs into exosomes by binding to specific motifs^27^. Zhang et al.^28^ concluded that mutant KRAS in CRC cells significantly change exosome composition, which promotes the malignant potential of recipient cells and eventually promote cancer progression. Mutant KRAS not only transfers intracellular circRNAs to exosomes and regulates exosomal miRNAs, such as increasing the content of miR-100 in exosomes, but also increase the levels of some tumor-promoting proteins, such as KRAS and EGFR, in exosomes^29–31^.

Exosomes carry a variety of proteins, some of which are intrinsic to exosomes, such as CD9, CD63, CD81, TSG101, and ALIX, and some of which represent the cellular origin of exosomes, thus making exosomes potential biomarkers^32^. For example, exosomal proteomic analysis of CRC revealed multiple exosomal protein markers, including vaccinia virus antigen A33, epidermal growth factor receptor (EGFR), and epithelial cell adhesion molecule (EpCAM)^33,34^. Indeed, existing evidence suggests that EpCAM is a CRC-derived exosome-specific protein^32^. A recent study showed that protein expression in urinary exosomes of CRC patients reflects the characteristics of tumorigenesis. Exosomes were isolated from the urine of CRC patients and healthy adults. The exosomes from these two sources had spherical membrane structures, 30–100 nm in size, and expressing CD9, CD63, and CD81. The expression of CEACAM7 and CEACAM1, however, were increased and the expression of CHMP4A, CHMP4B, CHMP2A, CHMP2B, and CHMP1B were decreased in exosomes derived from the urine of CRC patients compared to healthy controls^35^. Moreover, although the expression of CD63 in CRC-derived exosomes was not specific compared to normal controls, CD63 expression in CRC-derived exosomes was significantly higher than normal controls^36^. Taken together, these findings contribute to distinguishing tumor-derived exosomes from normal exosomes.

### Release of exosomes

As mentioned earlier, exosomes refer to ILVs that are released outside the cell when MVBs fuse with the plasma membrane; however, MVBs that do not fuse with the plasma membrane are degraded *via* fusion with lysosomes or autophagosomes^15,37^. The factors influencing the ultimate fate of MVBs can be divided into intrinsic and extrinsic factors. Intrinsic factors, such as the RAB and soluble N-ethyl maleimide sensitive factor attachment protein receptor (SNARE) families, are mainly directly involved in the biosynthesis and release of exosomes. Rab27a promotes MVB docking by preventing Coronin1b localization to invadopodia, antagonization of cortactin and actin disassembly, and ultimately promoting exosome secretion^38^. Small GTPases control the transport and secretion of intracellular secretory MVBs by modifying the cytoskeleton and locating vesicles on the cell plasma membrane^39^. Exosomal release is an energy-consuming process. Several protein-lipid and protein–protein interactions have been shown to reduce energy barriers and promote MVB fusion with cell membranes. For example, Rab31 recruits the GTPase-activating protein (GAP), TBC1D2B, which inhibits Rab7, leading to increased MVB membrane fusion and exosome secretion^40^. Arl8b can initiates the Arl8b/SKIP/HOPS cascade to recruit the GAP TBC1D15, which also inhibits Rab7^41^. SNARE proteins also promote the fusion of vesicles with the target membrane, such as the cell membrane or membranes of different organelles^42^. In addition, the microtubule cytoskeleton is also able to influence the transport of MVBs to the plasma membrane^38,43^.

External factors, such as acidity and calcium, are mainly cell microenvironment factors that affect the release of exosomes^44^. At present, many factors affecting exosome biogenesis and release have been reported; however, these reports are only the tip of the iceberg because the underlying mechanism has not been clarified. Cell type and cell homeostasis also have an important role in regulating the biogenesis and release of exosomes. In addition, it has been shown that different MVB subtypes use different pathways to fuse with the plasma membrane to release exosomes, which warrants further study.

### Absorption of exosomes

Exosomes can be taken up by recipient cells through various mechanisms, such as direct fusion with target cell membranes, uptake by target cells through endocytosis, and recognition of specific cell surface receptors^45^.

### Isolation and detection of exosomes

Because exosomes carry molecules with diverse biological functions and have great potential in the diagnosis of tumors, the technology enabling the isolation and detection of exosomes has also developed rapidly. Currently, in addition to the most common ultracentrifugation method, there are many other methods for the isolation of exosomes, including particle size exclusion chromatography, density gradient centrifugation, ultrafiltration centrifugation, the microfluidic chip method, the magnetic bead immunoassay, and the polymer precipitation method^46–49^. The purity and quantity of exosomes are the basis for their efficient detection. Compared with the traditional Western blot and ELISA-based molecular characterization, researchers have developed multiple more sensitive and specific detection methods, including chemiluminescence, electrochemical immunosensors, optical detection based on microfluidic chips, and dynamic light scattering^50–54^. In addition, surface-enhanced Raman scattering (SERS), which is based on functional metal nanoparticles, has attracted widespread attention in the detection of exosomes owing to its high sensitivity and capability of multiplex detection^55^. Diao et al.^56^ achieved discrimination of exosomes from healthy cells and cancer cells using a machine learning-based, label-free, SERS-sensing technique. In addition, Lin and colleagues^57^ designed a porous-plasmonic SERS chip functionalized with synthesized CP05 polypeptide that specifically captures and distinguishes exosomes from different sources. Based on the SERS chip, Lin and colleagues^57^ successfully distinguished lung and colon cancer cell-derived exosomes from healthy exosomes at the single vesicle level using unique Raman spectroscopy and machine learning methods.

**Figure 1:**
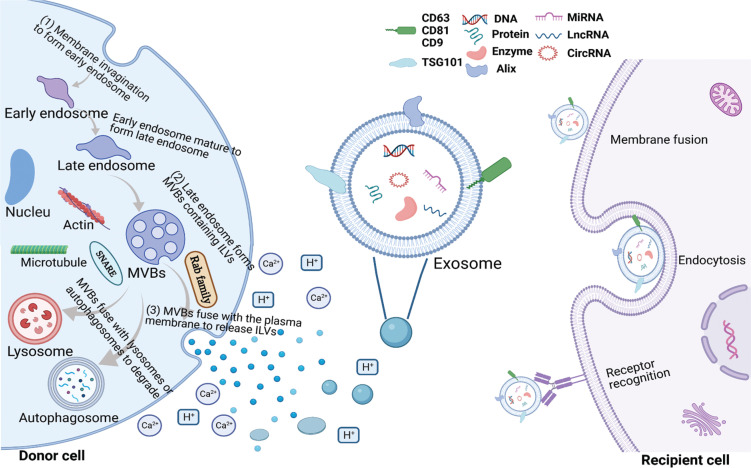
Biogenesis of exosomes in donor cells and absorption by recipient cells. Exosomes are involved in cell communication by carrying a variety of bioactive molecules, including DNA, proteins, enzymes, miRNAs, lncRNAs, and circRNAs. The biogenesis of exosomes is divided into three sequential stages in donor cells, which are annotated in the upper figure and mainly involve two sorting mechanisms (the ESCRT and ESCRT-independent pathways). The release of exosomes is also influenced by extracellular environmental factors, such as acidity and calcium. Exosomes can be absorbed by recipient cells through diverse mechanisms, such as a direct fusion with target cell membranes, uptake by target cells through endocytosis, and recognition of specific receptors on the cell surface.

The biosynthesis and release of exosomes is a multi-step dynamic process that is affected by many factors inside and outside the cell (**Figure 1**).

## Immunoregulatory role of exosomes in the CRC microenvironment

The TME is the environment in which tumors occur and develop. In addition to tumor cells, the TME includes other cell types, such as immune cells, mesenchymal stem cells (MSCs), and cancer-associated fibroblasts (CAFs)^58^. The crosstalk between tumor and immune cells mediated by exosomes is very active and promotes tumor progression within the TME^59^. Understanding the role of exosomes in immune regulation will help further clarify the specific mechanisms underlying CRC progression.

### Exosomes and tumor-associated macrophages (TAMs)

Macrophages are one of the most important immune cells and are mainly produced by monocytes migrating from peripheral blood to tissues^60,61^. TAMs are a group of macrophages that infiltrate tumor tissues^62^. The TME is complex and TAMs not only kill tumors, but also promote tumor growth^63^. Generally, macrophages are divided into M1- and M2-type macrophages based on surface molecule expression and functional differences. M1-type macrophages, also known as classically activated macrophages, are mainly induced by IFN-γ, a bacterial endotoxin (lipopolysaccharide), and cytokines, such as TNF and GM-CSF. M2 macrophages, which are referred to as activated macrophages, are mainly induced by interleukin (IL)-4, IL-13, IL-10, CSF-1, IL-1R ligands, and glucocorticoids^64–67^. M1 macrophages expressing high levels of MHCII and B7 molecules have a strong antigen-presenting capacity and secrete high levels of cytokines (IL-12, IL-23, IL-1, IL-6, IL-13, and TNF) and chemokines [CCL2 (MCP-1), CCL3 (MIP-1α), CXCL9 (MIG), and CXCL10 (IP-10)], thus activating the Th1-type immune response. M1 macrophages kill tumor cells and eliminate intracellular microorganisms by secreting high levels of cytotoxic intermediates, such as nitric oxide (NO) and reactive oxygen intermediates. Therefore, M1 macrophages are generally believed to have immune defense and antitumor roles. In contrast, the antigen-presenting function of M2 macrophages is low. M2 macrophages secrete high levels of the chemokines, CCL17 and CCL22, express high levels of the mannose, scavenger, and galactose receptors to inhibit the inflammatory response and Th1 cell-mediated acquired immune response, and promote wound healing, angiogenesis, and tissue repair. The M1 and M2 phenotypes represent the two extreme states of macrophages; however, macrophage activation is a complex process and macrophages exist in a spectrum of functional states^68–72^.

Numerous studies have shown that CRC-derived exosomes carry specific molecules to promote the differentiation of macrophages into the M2 phenotype, thereby mediating malignant tumor metastasis. Recently, a study showed that CRC cells that have undergone EMT promote M2-like macrophage polarization by significantly increasing the levels of miRNA-106b-5p (miR-106b) in macrophages *via* direct transfer of exosomes. Increased miR-106b levels directly inhibit programmed cell death 4 (PDCD4) at the posttranscriptional level to activate the phosphatidylinositol 3-kinase (PI3K)γ/AKT/mammalian target of rapamycin (mTOR) signaling cascade, thus contributing to M2 polarization of macrophages. Furthermore, activated M2 macrophages promote EMT-mediated migration, invasion, and metastasis of CRC cells in a positive feedback manner^73^. Several miRNAs from CRC cell-derived exosomes, including miR-934, miR-25-3p, miR-130b-3p, and miR-425-5p, have been shown to induce M2 macrophage polarization through activation of the PI3K/AKT signaling pathway^74,75^. M2-polarized macrophages secrete the chemokine, CXCL13, and activate the CXCL13/CXCR5 axis in CRC cells, thus promoting metastasis to the liver. In addition to miRNAs, lncRNA RPPH1 from CRC-derived exosomes can be transferred to macrophages, mediating polarization into the M2 phenotype and thereby promoting the proliferation and metastasis of CRC cells^76^. Like tumor cells, macrophages can also secrete exosomes and exert a regulatory role within the TME. *In vivo* and *in vitro* studies have confirmed that M2 macrophage-derived exosomes are rich in miR-21-5p and miR-155-5p, and promote the migration and invasion of CRC cells. The specific mechanism has been reported to involve miR-21-5p and miR-155-5p binding to the coding sequence of BRG1 after being transferred into CRC cells^77^; however, it has also been reported that CRC-secreted exosomes carrying specific molecules have the potential to skew macrophages toward an anticancer M1-like phenotype^78^.

### Exosomes and myeloid-derived suppressor cells (MDSCs)

MDSCs, which are derived from bone marrow progenitor and immature myeloid cells, are widely present in the peripheral blood and tumor tissues of patients^79,80^. MDSCs normally differentiate into dendritic cells (DCs), macrophages, and granulocytes, but significant increases in the number and proportion of MDSCs have been observed in tumor tissues and are correlated with tumor size and malignancy^81,82^. Granulocyte MDSCs enhance stemness and growth of CRC cells by secreting exosomal S100A9 in CRC patients^83^. Tumor-derived exosomes associated with Hsp72 trigger Stat3 phosphorylation in MDSCs in a TLR2/MyD88-dependent manner *via* autocrine IL-6 signaling, thus enhancing the immunosuppressive function of MDSCs^84^.

### Exosomes and neutrophils

Neutrophils are the first line of defense against foreign pathogens and an important component of the immune system^85^. Interestingly, neutrophils not only infiltrate tumor tissues, but also have a dual role in promoting or inhibiting tumor malignant progression under the effects of different factors within the TME^86^. Studies have confirmed that neutrophils within the TME differentiate into an anti-tumor N1 phenotype and a pro-tumor N2 phenotype in response to different stimuli^87–89^. A growing number of studies have shown that exosomes regulate the interaction between tumor cells and neutrophils within the TME. CRC-secreted exosomes containing circPACRGL promote the proliferation, migration, and invasion of CRC cells by regulating the miR-142-3p/miR-506-3p-TGF-β1 axis. TGF-β1 overexpression in the TME promotes the transformation of N1 neutrophils to N2 neutrophils, which can promote tumor proliferation and metastasis, and may explain this phenomenon^90^. Exosomes carrying KRAS mutants transfer these mutated forms to recipient cells, thus promoting neutrophil recruitment in the CRC microenvironment and the formation of neutrophil extracellular traps by upregulating IL-8 expression, which leads to a decrease in CRC tumor size^91^. *In vivo* studies have shown that exosomes secreted by CRC stem cells (CRCSCs) are transported to the bone marrow, which can prolong the survival time and activate the protumor phenotype of bone marrow-derived neutrophils. Tumor exosomal triphosphate RNAs induce the expression of IL-1β *via* the NF-κB signaling axis to sustain neutrophil survival^92^.

### Exosomes and natural killer (NK) cells

NK cells are an important component of the innate immune system, and can kill tumor cells by directly acting or secreting cytokines and other substances^93,94^. Numerous studies, however, have shown that NK cell function is inhibited or abnormal within the TME, which contributes to tumor immune escape^95^. Specifically, the proportion of NK cells is significantly reduced in patients with esophageal squamous cell carcinoma, which mediates tumor immune escape^96^. As an important communicator of intercellular information, exosomes also play a crucial role in the crosstalk between tumors and NK cells. HCC cells secrete exosomal circUHRF1 to upregulate TIM3 expression in NK cells, which leads to an exhaustion and reduction in the level of NK cell infiltration in tumor tissues, which ultimately promotes tumor development^97^. CRC cells transport lncRNA SNHG10 into NK cells through exosomes, which mediates the upregulation of INHBC expression and activation of the TGF-β signaling pathway in NK cells, thereby inhibiting the cytotoxic effect of NK cells and promoting CRC immune escape^98^.

NK cell-based therapeutic strategies have shown promise in hematologic malignancies^99^; however, due to a poor ability to infiltrate solid tumors and the role of inhibitory signals within the TME, the efficacy of targeting NK cells in the treatment of solid tumors is not satisfactory. Nevertheless, studies have shown that NK cells are able to release EVs containing cytolytic proteins, suggesting that NK cell-derived EVs may have therapeutic potential. One study showed that EVs secreted by IL-12/15/18-activated primary NK cells or the NK cell line, NK-92, have a greater ability to induce apoptosis in colon cancer cells than IL-15-stimulated primary NK cells or NK-92. The study pointed to NKG2D as the receptor mediating the interaction between NK cell-derived EVs and HCT116 spheroids. HCT116 cells express high levels of the NKG2D ligand, MICA/B, and the susceptibility of tumor tissues to NK cell-derived EVs is associated with the differential expression of NKG2D ligand MICA/B, which can be blocked by anti-NKG2D antibodies^100^. These results suggest that NK cell-derived EVs have the potential to target solid tumors. Interestingly, exosomes secreted by tumor cells have been shown to induce the killing activity of NK cells. For example, Hsp70/Bag-4 surface-positive exosomes secreted by Hsp70/Bag-4 positive pancreas (Colo+) and colon (CX+) carcinoma sublines induced NK cell migration and killing activity that was completely abolished by Hsp70-specific antibodies^101^. These results suggest that targeting NK cells with engineered exosomes can be used as a strategy for cancer therapy.

### Exosomes and DCs

DCs efficiently take up, process, and present antigens and are considered the most powerful antigen-presenting cell (APC) in the body^102,103^. In general, immature DCs have a strong migration ability, while mature DCs, an effective initiator of the adaptive immune response, effectively activate naive T cells^104^. In recent years, studies have shown that DC-derived exosomes contain abundant MHC class I and II molecules, as well as T cell co-stimulatory molecules, that can effectively trigger the induction and expansion of adaptive immune responses in lymphoid organs^105^. In addition, after effectively ingesting the natural tumor antigens in exosomes derived from tumor cells, DCs process these tumor antigens and present the tumor antigens to cytotoxic T lymphocytes (CTLs) to induce anti-tumor responses. Therefore, an increasing number of studies have shown that exosomes secreted by DCs and tumor cells can be used as a cell-free vaccine to induce effective anti-tumor immunity and serve as an important approach to treat tumors^105^.

Fibroblast activation protein-α (FAP) gene-modified exosome-like nanovesicles (eNVs-FAP) derived from tumor cells have shown good anti-tumor effects in multiple tumor-bearing mouse models, including colon cancer. DCs present antigens from eNVs-FAP, which activate antigen-specific CD8^+^ T cells to kill FAP^+^CAFs and tumor cells. Moreover, eNVs-FAP alleviate immunosuppression in the microenvironment and promote the immune response by promoting the transformation of M2 TAMs to M1 TAMs and eliminating immunosuppressive MDSCs and regulatory T cells (Tregs)^106^. Exosomes derived from DCs infected with the Me49 strain of *Toxoplasma gondii* (DC-Me49-exo) have been shown to inhibit the growth of CRC in a mouse model. DC-Me49-exo was also shown to exert an anti-tumor effect mainly by inhibiting the STAT3 signaling pathway to reduce the proportion of MDSCs. Pathogen infection and tumor “infection” may compete to regulate the immune system. Exosomes isolated from *T. gondii*-infected DCs can act as messengers to stimulate the immune system and turn “cold” tumors into “hot” tumors. DC-Me49-exo may be a promising therapeutic strategy to inhibit CRC progression^107^. Cancer stem cells (CSCs) are an important cause of treatment resistance and recurrence of CRC. Therefore, the development of therapeutic strategies against CSCs is a major challenge in current cancer treatment. Naseri et al.^108^ reported that DCs loaded with exosomes derived from CSC-enriched colonospheres (CSCenr-EXOs) promote the ability of T lymphocytes against colorectal CSCs *in vitro*. These results suggested that DCs loaded with CSCenr-EXOs could be used as a novel antigen source to stimulate T cell proliferation and cytotoxicity of targeting CSCs^108^.

### Exosomes and B lymphocytes

B lymphocytes, also known as B cells, mature in the bone marrow and migrate through the blood to the peripheral lymphoid organs. Mature B cells are activated in response to antigen stimulation, thereby synthesizing and secreting antibodies to mediate humoral immune responses^109^. There are a variety of membrane surface molecules on the surface of B cells that recognize antigens, interact with other molecules and cells, and participate in immune regulation and the inflammatory response^110^.

Numerous studies have shown that tumor growth is not only related to cellular immunity, but also closely related to humoral immunity. B cells have a key role in humoral immunity and are an important component of lymphocyte infiltration in multiple tumors, including CRC^111–113^. Regulatory B cells (Bregs), a subgroup of B cells, highly express PDL1 and participate in tumor immunosuppression, as well as promote tumor development^114^. Several studies have shown that compared with tumor cells, PDL1 on host immune cells, especially APCs, is more important in immune checkpoint blockade therapy^115,116^. Exosomes, as message carriers, have an important role in remodeling the TME. Xie et al.^117^ recently showed that CRC-derived exosomes drive Breg-suppressed anti-tumor immune processes by delivering the lncRNA, HOTAIR. CRC-derived HOTAIR promotes the polarization of B cells to PDL1-tagged Bregs and induces PDL1^+^ B cells to suppress the activity of CD8^+^ T cells. Exosomal HOTAIR binds to pyruvate kinase M2 (PKM2) and protects PKM2 from ubiquitination degradation, which leads to STAT3 activation and PDL1 expression. In addition, a study involving CRC patients showed that exosomal HOTAIR is positively correlated with infiltration of PDL1^+^ B cells in tumor tissues, suggesting that exosomal therapy targeting HOTAIR may be a new therapeutic strategy for CRC.

### Exosomes and T lymphocytes

T lymphocytes, a group of lymphoid stem cells derived from the bone marrow, are a key component of the body’s immune response. T lymphocytes not only have roles in infections, allergic diseases, and autoimmune diseases, but also have an immunomodulatory role in the antitumor process^118,119^. Active T lymphocyte infiltration in neoplastic lesions is often associated with a better prognosis. The presence of T cells in CRC tissues attenuates the metastatic potential of tumor cells and is inversely associated with tumor recurrence after treatment^120,121^. This pattern has also been observed in several other malignancies, such as ovarian cancer and melanoma^122,123^. CD8^+^ CTLs are the main force in the antitumor immune response. Activated CTLs kill tumor cells by releasing perforin and granzyme to cause necrosis of target cells or by inducing apoptosis of target cells *via* surface molecules^124^. CD4^+^ T cells (Th cells) mainly exert antitumor effects by assisting CD8^+^ T cells and B cells^125^; however, the tumor immune microenvironment is quite complex and contains not only various antitumor immune components, but multiple immunosuppressive components. Tregs are an immunosuppressive subset of Th cells and have an important role in the malignant progression of CRC^126,127^.

Some evidence suggests that exosomes regulate T lymphocyte function^128^. A recent study showed that infusion of reovirus in combination with FOLFIRI plus bevacizumab treatment significantly reduced the level of serum exosomal miR-29a-3p in CRC patients with a KRAS mutation. This treatment resulted in enhanced activity of CD8^+^ and CD4^+^ T lymphocytes^129^. Tumor-derived exosomal PD-L1 binds to the PD-1 receptor on T cells in draining lymph nodes and inhibits the activity of T cells, thus promoting the malignant progression of CRC^130^. Mima et al.^131^ reported that the expression of miR-21 in CRC cells is negatively correlated with the density of infiltrating T cells and that miR-21 has a regulatory role in inhibiting the adaptive immune response mediated by antitumor T cells, suggesting that miR-21 may be a potential target for CRC immunotherapy and prevention. Many studies have also shown that miR-21 is upregulated in CRC-derived exosomes and is involved in the malignant progression and drug resistance of CRC^132,133^. Moreover, a recent study revealed that miR-21-5p and miR-200a in CRC-derived small EVs synergistically induced macrophage M2-like polarization and PD-L1 expression, thus leading to decreased CD8^+^ T-cell activity and accelerated tumor growth^134^. At present, there are relatively few reports focusing on the interaction between exosomes and T lymphocytes. Given that T lymphocytes are key to the body’s immune response, further studies are warranted.

### Exosomes and CAFs

CAFs are the main components of stromal cells within the TME, and CAFs participate in many aspects of tumor development^135^. Numerous studies have shown that CAFs have a role in mediating tumor development by secreting cytokines and EVs, including exosomes, thus regulating the composition of ECM or by direct contact with tumor cells^136^. Specifically, CAFs mediate tumor angiogenesis, proliferation, tumor metastasis, and treatment-resistant tumors^137^. For example, CAF-derived exosomal miR-181b-3p significantly reduces apoptosis and enhances the proliferation and migration of CRC cells by regulating the expression of SNX2 in CRC cells^138^. Similarly, Shi et al.^139^ reported that CAFs transport miR-345-5p to CRC cells *via* exosomes, thereby promoting CRC progression. The underlying mechanism involves exosomal miR-345-5p promotion of CRC cell growth and metastasis by inhibiting CDKN1A expression in CRC cells. CAF-derived exosomes promote growth of CRC and angiogenesis by upregulating miR-135b-5p expression and inhibiting thioredoxin-interacting protein expression in CRC cells^140^. In addition to miRNAs, lncRNAs in the exosomes secreted by CAFs also regulates CRC progression. The exosomal lncRNA, LINC00659, which is secreted by CAFs, promotes the proliferation and metastasis of CRC cells by directly interacting with miR-342-3p to increase ANXA2 expression^141^. Expression of the lncRNA, WEE2-AS1, was increased in CAF-derived exosomes compared with normal fibroblast (NF)-derived exosomes. WEE2-AS1 in exosomes can be taken up by CRC cells and promotes the progression of CRC. WEE2-AS1 functions as a modular scaffold for the MOB1A and E3 ubiquitin-protein ligase praja2 complexes, thus leading to MOB1A degradation and Hippo pathway inhibition^142^. In addition, CAFs mediate the development of drug resistance in CRC cells. For example, a recent study revealed that CAFs promote cisplatin resistance and malignant progression in CRC cells. CAFs extracted from tissue samples of cisplatin-resistant CRC patients deliver VEGFA to HT29 cells *via* exosomes, thus promoting cells resistance to cisplatin, enhancing cell viability, reducing cell apoptosis, and promoting angiogenesis^143^. Exosomes have been shown to mediate CAF regulation of radiotherapy resistance in CRC. CAF-derived exosomes downregulate CLCA4 expression, induce inactivation of PI3K/Akt signaling pathway by delivery of miR-590-3p to CRC cells, and mediates radioresistance of CRC cells^144^. In addition, Liu et al.^145^ reported that CAF-derived exosomes regulate radioresistance by promoting the CRC stemness phenotype. Taken together, it has been shown that CAF-derived exosomes promote CRC progression, as well as treatment resistance.

The interaction between CRC cells and CAFs is reciprocal and dynamic within the TME. CAFs influence tumor cell progression through a variety of mechanisms. Similarly, tumor cells mediate the formation of CAFs by promoting the activation of NFs into CAFs. MiR-146a-5p and miR-155-5p can be delivered to fibroblasts by exosomes from CXCR7-overexpressing CRC cells and promote the activation of fibroblasts to CAFs *via* the JAK2-STAT3/NF-κB signaling pathway by inhibiting the expression of SOCS1 and ZBTB2. In turn, activated CAFs further trigger EMT and the pro-metastatic switch of CRC cells and enhance the invasive ability of CAFs. Moreover, CAFs activated by miR-146a-5p and miR-155-5p promote tumorigenesis and lung metastasis of CRC *in vivo*^146^. Rai et al.^147^ found that exosomes derived from both the early-stage CRC cells (SW480) and late-stage CRC cells (SW620) activated NFs. Moreover, the results showed that SW480 cell-derived exo-activated fibroblasts were highly pro-proliferative and pro-angiogenic, while SW620 cell-derived exo-activated fibroblasts showed a striking ability to invade through the extracellular matrix (ECM) by upregulating pro-invasion regulators of membrane protrusion and matrix-remodeling proteins based on proteomic and functional analyses. This study emphasized the role of primary and metastatic CRC-derived exosomes in generating phenotypically and functionally distinct subsets of CAFs that contribute to tumor progression. In addition, numerous studies have revealed that CAFs are a highly-heterogeneous subset with different tumor-promoting effects and tumor-suppressing functions^148^. The reason for this phenomenon may be that the sources of CAFs are diverse. In addition to the transformation of NFs in the tissues, CAFs can also be differentiated from epithelial cells, endothelial cells, and bone marrow MSCs in tumor tissues. In contrast, the highly-heterogeneous CAF subsets may reflect the dynamic and complex TME. Similar to the temporal and spatial heterogeneity of tumors, CAFs in different stages or different tumor tissues may be affected by different factors, and thus have diverse phenotypes.

### Exosomes mediate crosstalk between immune cells and CRC with different immune status

Compared with the microsatellite stability (MSS)/microsatellite instability (MSI)-low CRC, the MSI-high (MSI-H) CRC has more immune cell infiltration, higher immune-related gene expression, and higher immunogenicity^149^. It has been shown that exosomes mediate crosstalk between immune cells and MSI/MSS CRC, and participate in the regulation of CRC immune status and function in the TME^149^. Cui et al.^150^ identified three exosome-related genes (ERGs) as key prognostic genes in CRC patients through bioinformatics analysis and confirmed that these ERGs were highly expressed in multiple CRC cells. Cui et al.^150^ calculated risk scores for CRC patients based on the coefficients of these ERGs and divided patients into high- and low-risk groups. Further analysis revealed that the levels of resting CD4 memory T cells, resting dendritic cells, and activated dendritic cells were higher in patients with low-risk scores, while the level of activated NK cells was higher in patients with a high-risk score. Genes associated with DNA mismatch repair (MMR) were upregulated in the low-risk group. In addition, the high-risk group had more MSI-H patients than the low-risk group, and the MSI-H patients had higher risk scores than the MSI low (MSI-L) and MSS patients. EVs containing miR-424 secreted by CRC cells inhibited CD28-CD80/86 co-stimulatory pathways in tumor-infiltrating T cells and dendritic cells, thus leading to immune checkpoint blockade resistance. Moreover, CRC-derived EVs with miR-424 knocked down enhanced the T-cell-mediated antitumor immune response in CRC models^151^.

**Figure 2:**
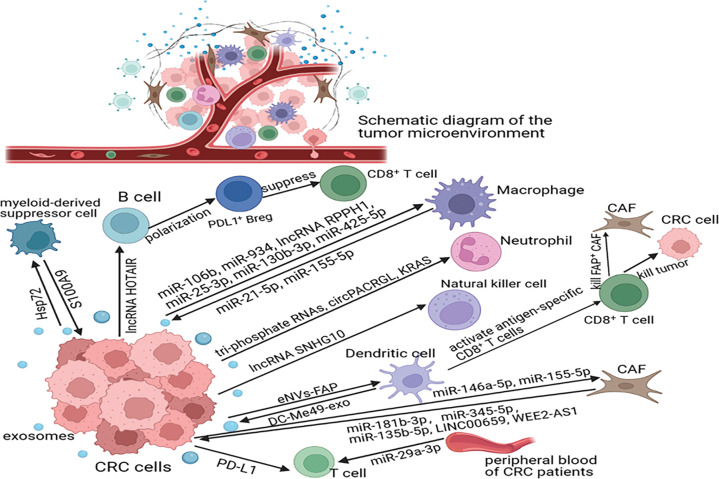
Key molecules involved in exosome crosstalk between CRC and immune cells. CRC cells influence macrophage polarization as follows: releasing exosomes carrying specific molecules, such as miRNAs or lncRNAs; affecting neutrophil function or differentiation *via* exosomal triphosphate RNAs, circPACRGL, or KRAS; enhancing the immunosuppressive function of MDSCs *via* exosomal Hsp72; promoting the polarization of B cells to PDL1^+^ Breg *via* exosomal lncRNA HOTAIR; affecting the killing ability of NK cells *via* exosomal lncRNA SNHG10; suppressing T-cell function *via* exosomal PD-L1 or miR-29-3p; and mediating CAF activation. Furthermore, some molecules that are carried by immune cell-derived exosomes, including miR-21-5p, miR-155-5p, S100A9, miR-181b-3p, miR-345-5p, miR-135b-5p, linc00659, and WEE2-AS1, promote the migration and invasion of CRC cells.

The crosstalk between CRC and the immune system is dynamic and complex and has a vital role in CRC progression (**Figure 2**).

## Regulatory role of exosomes in CRC metastasis

Metastasis is the leading cause of death in cancer patients^152^. Exosomes have a crucial role in tumor metastasis by conveying specific information between cells^153^. Numerous studies have shown that exosomes are involved in multiple processes of tumor metastasis, including epithelial-mesenchymal transformation (EMT), tumor angiogenesis, ECM remodeling, and premetastatic niche (PMN) formation^154,155^. In addition, exosomes also participate in regulation of intestinal flora in CRC metastasis^156^.

### Exosomes mediate EMT in CRC metastasis

EMT is an important biological process in which tumor cells acquire the ability to migrate and invade^157^. Therefore, elucidating the molecular mechanism underlying regulation of the EMT process is significant in the control and prevention of tumors. Although the mechanism underlying the EMT in CRC is not clear at present, growing evidence has revealed that exosomes mediate the EMT process of CRC in the microenvironment. CAFs are key components of the TME and have multiple functions, including matrix deposition and remodeling, and extensive crosstalk with tumor cells^158,159^. A study has shown that the lncRNA, LINC00659, is significantly expressed in CAFs-derived exosomes and promotes the EMT process of CRC after transfer to CRC cells *via* exosomes^141^. Liu et al.^160^ extracted exosomes from the serum of CRC patients for RNA-seq, and found that exosomal miR-106b-3p was highly expressed in the serum of patients with CRC metastasis. Further studies showed that exosomal miR-106b-3p promoted the EMT process and invasion of CRC by directly targeting DLC-1, and ultimately promoted CRC lung metastasis. A recent study revealed higher levels of FZD10 expression in exosomes secreted by the metastatic CRC cell line, SW620, compared to the non-metastatic CRC cell line, CaCo-2. SW620-derived exosomal FZD10 significantly promotes the expression of c-myc and vimentin, which are markers of EMT, in recipient cells^161^. Hypoxia induces EMT in tumor cells. Interestingly, one study showed that hypoxia induced changes in exosomal miRNAs secreted by CRC cells, which in turn influenced tumor cell proliferation and EMT. Hypoxia directly reduces the amount of exosomal miR-1255b-5p secreted by CRC cells, thereby enhancing EMT and telomerase activity by increasing hTERT expression. This conclusion was also validated in a mouse CRC model, in which exosomes overexpressing miR-1255b-5p were shown to impair EMT and liver metastasis *in vivo*^162^.

### Exosomes mediate angiogenesis in the metastasis of CRC

Angiogenesis is the core event in malignant tumor progression. Numerous studies have confirmed that exosomes regulate angiogenesis in multiple tumors, including thyroid carcinoma, gastric cancer, lung cancer, and CRC^163–166^. Hu et al.^167^ reported that CRC-derived exosomal miR-1229 promoted tube formation and migration of HUVECs, and further studies showed that exosomal miR-1229 activated the VEGF pathway by inhibiting HIPK2 expression, thus exhibiting a pro-angiogenesis role. Additionally, a study showed that miR-25-3p can be transferred from CRC cells to endothelial cells *via* exosomes. Exosomal miR-25-3p regulates the expression of VEGFR2, ZO-1, occluding, and claudin5 in endothelial cells by targeting KLF2 and KLF4, thus promoting vascular permeability. Furthermore, exosomal miR-25-3p from CRC cells dramatically induces vascular leakage and promotes CRC metastasis to the liver and lungs of mice^168^. Within the complex TME, exosomes do not always have a role in promoting tumor angiogenesis; indeed, the role mainly depends on the molecules exosomes carry. For example, a recent study reported that exosomal circFNDC3B inhibits CRC angiogenesis^169^.

### Exosomes mediate ECM remodeling

The ECM is a non-cellular three-dimensional macromolecular network composed of collagens, proteoglycans/glycosaminoglycans, elastin, fibronectin, laminins, and several other glycoproteins, and is involved in the malignant progression of tumors^170^. Many studies have shown that tumor cells secrete exosomes that mediate ECM remodeling to create favorable conditions for metastasis. Exosomes derived from colon cancer alter the phenotype and function of fibroblasts and macrophages, thus causing changes in the composition of the ECM, which contributes to the migration and invasion of colon cancer^171^. Fibroblasts can increase the secretion of matrix-remodeling proteins, including MMP11, EMMPRIN, and ADAM10, after activation by CRC cell SW620-derived exosomes^147^. CRC-secreted exosomal miR-1246 participates in the degradation of ECM within the TME. The specific mechanism involved CRC-derived exosomal miR-1246 reprogramming macrophages into TAMs, which can cause degradation of the ECM^172^.

### Exosomes mediate the formation of PMN

The PMN is a microenvironment conducive to metastasis created by primary tumors in secondary organs and tissues^173^. PMN is mainly composed of tumor-derived factors, bone marrow-derived cells recruited by tumors, and stromal cells at metastatic sites. Exosomes, as the main components of tumor-derived factors, mediate material exchange and signaling between various cells in the PMN. The exosomal miR-21-5p secreted by CRC cells induces liver macrophages to polarize toward a pro-inflammatory phenotype and secrete IL-6 by downregulating the expression of Toll-like receptor 7, thus establishing an inflammatory PMN conducive to liver metastasis^174^. Zhang et al.^175^ demonstrated that CRC-derived exosomal HSPC111 promoted CRC liver metastasis by promoting PMN formation in a xenograft mouse model. Exosomal HSPC111 activated fibroblasts into CAFs and phosphorylated ATP-citrate lyase to increase acetyl-CoA levels in CAFs. Moreover, the increased secretion of CXCL5 in CAFs reinforced exosomal HSPC111 secretion and promoted the metastasis of CRC *via* the CXCL5-CXCR2 axis. In addition, TGF-β contained in CRC-derived exosomes promoted the formation of PMNs by activating fibroblasts into CAFs^176^. MiR-221/222 secreted by CRC can be transported to liver stromal cells *via* exosomes to induce the formation of PMNs, thus providing a suitable colonization environment for incoming metastatic tumor cells and consequently contributing to CRC metastasis^177^.

### Exosomes mediate the regulation of intestinal flora in CRC metastasis

In recent years the role of gut microbiota in the development of gastrointestinal tumors has attracted extensive attention. For example, adverse microbiota, metabolites, and cytokines promote CRC metastasis by promoting the formation of a favorable microenvironment at both local and distant metastatic sites^178^. Direct supplementation of dietary fiber and probiotics or changing the structure of intestinal flora by fecal microbiota transplantation increase the level of short-chain fatty acids, thereby inhibiting tumor development^179,180^. Evidence suggests that exosomes participate in CRC metastasis mediated by intestinal microbiota. *Fusobacterium nucleatum* is an important intestinal bacterium. Guo et al.^181^ reported that *F. nucleatum* stimulates tumor cells to secrete exosomes enriched in miR-1246, miR-92b-3p, miR-27a-3p, and CXCL16. These exosomal components regulate the biological activity of uninfected cells by activating the Wnt/β-catenin signaling pathway and downregulating GSK3β expression, ultimately creating a favorable environment for CRC proliferation, invasion, and migration.

## Potential of exosomes as biomarkers and the therapeutic significance in CRC

Because classical EVs are wrapped in double-layer phospholipid membranes, exosomes contain a variety of functional bioactive molecules that are involved in multiple processes, such as tumorigenesis, metastasis, drug resistance, and CRC therapy. With the advantages of stability and the ability to carry abundant information, exosomes have shown great prospects in clinical applications.

### Potential exosomal biomarkers for CRC diagnosis and companion diagnostics

Exosomes can be detected in various body fluids, such as blood, urine, saliva, and breast milk, thus having great potential as diagnostic markers for tumors^8,182^. For example, circulating exomiR-1229 levels are significantly increased in serum exosomes from CRC patients^167^. RNA sequencing of serum exosomes from healthy subjects and CRC patients confirmed the presence of many circRNAs, including circ-KLDHC10, has_circ_0010522 (circ-133), hsa_circ_0004771, hsa_circ_0067835 (circIFT80), circRHOBTB3, hsa-circ-0005100 (circFMN2), and circ-PNN (hsa_circ_0101802). These circRNAs were shown to have significantly higher levels in serum exosomes from CRC patients than healthy controls^183–189^. Notably, higher levels of exosomal molecules in CRC patients do not indicate that these exosomal cargos promote tumor progression^187^. Exosomal molecules that are upregulated or downregulated in serum exosomes from CRC patients compared with healthy subjects have the potential to be diagnostic markers for CRC. Receiver operating characteristic (ROC) curve analysis showed that circ-PNN has a significant value in CRC diagnosis, with area under the ROC curve (AUC) values of 0.855 and 0.826 in the training and validation sets, respectively, suggesting that exosomal circ-PNN may be a non-invasive biomarker for CRC diagnosis^189^. Exosomal hsa_circ_0004771 is also effective in distinguishing CRC patients from healthy individuals^185^.

Currently, tissue biopsy and subsequent genomic characterization are used to provide a precise diagnosis to guide treatment for the majority of tumor patients; however, because tumors exhibit spatial or temporal heterogeneity, tissue biopsy is subject to sample bias, with studies showing that up to two-thirds of mutations would not be detected in a single biopsy in the remaining sampling regions of the same tumor^190,191^. In addition, sampling difficulties can lead to insufficient tissue for biopsy or genetic testing in some tumor types. Indeed, many studies have shown that exosomes can be used as a companion diagnosis to determine which patients will benefit most from certain drugs^192,193^. For example, Kharmate et al.^194^ showed that EGFR exists in serum exosomes of prostate cancer patients, which is helpful for the companion diagnosis of cetuximab. BRAFV600E in EVs can be used as a companion diagnosis for melanoma patients receiving vemurafenib^195^.

Compared with plasma EVs derived from healthy controls, VEGF and CD133 constitute a unique CRC signature in plasma EVs of CRC patients, which can be used for the companion diagnosis of CRC patients treated with bevacizumab^196^. KRAS and BRAF are genes with significant clinical implications for prognosis and management of CRC. A study identified somatic BRAF and KRAS mutations circulating in plasma of mCRC patients directly from oil-encapsulated EVs for digital PCR, with 100% concordance with tissue diagnostics. Importantly, the study identified additional somatic alterations in 7% of wild-type CRC cases, which were subsequently validated by further examination in the matched tissue biopsies^197^. In addition, Lucchetti et al.^198^ evaluated the content of exosomes and KRAS mutation status in exosomal DNA in 70 mCRC patients and 29 primary CRC patients, and analyzed serial blood samples at different disease stages. Lucchetti et al.^198^ demonstrated a significant correlation between disease extent and the number of exosomes, and 91% of mutated mCRC patients became wild type after first-line chemotherapy, suggesting that plasma exosomal KRAS mutation status is predictive of prognosis in mCRC patients. In conclusion, companion diagnosis provides a minimally invasive means for clinical monitoring of patients, conducive to determining the treatment response and timely adjust the treatment plan in case of disease recurrence.

The potential exosomal biomarkers for CRC diagnosis and companion diagnostics are shown in **Table 1**.

**Table 1:** Functions of exosomes as potential biomarkers in CRC

Exosome molecules	Regulation	Major functions	References
miR-1229	↑	CRC diagnosis	^167^
circ-KLDHC10	↑	CRC diagnosis	^184^
hsa_circ_0004771	↑	CRC diagnosis	^185^
hsa_circ_0005100	↑	CRC diagnosis	^188^
hsa_circ_0101802	↑	CRC diagnosis	^189^
circRHOBTB3	↑	CRC diagnosis	^187^
has_circ_0010522	↑	CRC diagnosis	^183^
hsa_circ_0067835	↑	CRC diagnosis	^186^
CPNE3	↑	CRC diagnosis	^199^
VEGF and CD133	↑	Companion diagnosis receiving bevacizumab therapy	^196^
lncRNA UCA1	↑	Companion diagnosis receiving cetuximab therapy	^200^
FZD10	↑	Promoting EMT	^161^
LINC00659	↑	Promoting EMT	^141^
miR-1229	↑	Lymph node metastasis	^167^
miR-128-3p	↑	Tumor stage	^201^
miR-92a-3p	↑	Lung metastasis	^202^
miR-106b-3p	↑	Lung metastasis	^160^
HSPC111	↑	Liver metastasis	^175^
miR-25-3p	↑	Liver and lung metastasis	^168^
circLONP2	↑	Liver and lung metastasis	^203^
miR-193a	↓	CRC progression	^204^
let-7g	↑	CRC progression	^204^
miR-221/222	↑	CRC progression	^177^
hsa_circ_0000338	↑	Resistance to FOLFOX/5-FU	^205,206^
lncRNA HOTTIP	↑	Resistance to mitomycin	^207^
lncRNA H19	↑	Resistance to oxaliplatin	^208^
hsa_circ_0005963	↑	Resistance to oxaliplatin	^209^
lncRNA CCAL	↑	Resistance to oxaliplatin	^210^
miR-46146	↑	Resistance to oxaliplatin	^211^
miR-208b	↑	Resistance to oxaliplatin	^212^
p-STAT3	↑	Resistance to 5-FU	^213^
miR-21	↑	Resistance to 5-FU	^214,215^
miR-204-5p	↑	Enhancing sensitivity to 5-FU	^216^
miR-92a-3p	↑	Resistance to oxaliplatin/5-FU	^202^
Wnt	↑	Resistance to oxaliplatin/5-FU	^217^
CPT1A siRNA	↑	Reverse oxaliplatin resistance	^218^
Anti-miRNA-221 oligonucleotide	↑	Inhibit the proliferation and clonal formation of colon cancer cells	^219^
Doxorubicin	↑	Drug delivery	^220–222^

### Potential exosomal biomarkers for predicting CRC progression

Mounting evidence has shown that exosomes not only serve as markers for the diagnosis of CRC, but also have great potential for predicting CRC progression. Metastasis is the leading cause of death in all malignancies, including CRC. Patients with distant metastases in the liver, lung, and lymph nodes have a very low 5-year survival rate. Therefore, it is very important to quickly find effective indicators for the early detection and/or prognosis of metastasis-related conditions to improve the survival rate of CRC patients.

Increasing evidence indicates that bioactive molecules in exosomes, such as ncRNAs and proteins, are involved in distant metastasis of CRC. For example, LINC00659 is significantly elevated in CAF-derived exosomes and can be transferred to CRC cells to promote proliferation, invasion, migration, and EMT *in vitro*^141^. The level of exosomal miR-25-3p in the serum of CRC patients with metastasis was reported to be higher than the serum of CRC patients without metastasis, suggesting that miR-25-3p has enormous potential as a CRC metastasis blood marker^168^. Some molecules in exosomes from CRC patients, including miR-1229, miR-128-3p, miR-193a, let-7g, and miR-92a-3p, are associated with invasion, lymphatic metastasis, TNM stage, and survival rate^167,201,202,204^. Some exosomal molecules promote lung or liver metastasis of CRC. For example, the level of serum exosomal miR-106b-3p in CRC patients with metastasis was significantly higher than CRC patients without metastasis and experiments confirmed that exosomal miR-106b-3p promotes lung metastasis of CRC cells *in vivo*^160^. Another example involves the gene cluster, miR-221/222, which is upregulated in serum exosome samples from patients with CRC liver metastasis (CRLM); this upregulation was associated with lower overall survival^177^. In addition, CRC patients with liver metastasis have higher levels of HSPC111 in serum exosomes than CRC patients without liver metastasis, and HSPC111 promotes the formation of a premetastatic niche and CRLM by reprogramming lipid metabolism in CAFs, which suggests that HSPC111 may be a therapeutic target for the prevention of CRLM^175^.

The potential exosomal biomarkers for predicting CRC progression are shown in **Table 1**.

### Potential exosomal biomarkers related to CRC chemoresistance and drug-loading therapy

Although molecular targeted therapy and immunotherapy have made breakthrough progress in the treatment of CRC in recent years, chemotherapy is still an important treatment for advanced CRC patients. Unfortunately, many patients develop resistance to chemotherapy after a period of treatment, leading to a poor prognosis. Therefore, there is an urgency to identify a novel biomarker that can distinguish chemotherapy-resistant from -sensitive patients to accurately individualize the medication. It is also important to promptly elucidate the specific mechanism underlying chemotherapy resistance to find effective strategies for delaying or even avoiding the occurrence of drug resistance. Growing evidence indicates that exosomes have a role in mediating the occurrence and development of chemotherapy resistance in CRC patients.

Several studies have reported that exosomes mediate the resistance of CRC patients to oxaliplatin. CAF-derived exosomes can transfer lncRNA CCAL into CRC cells and increase the levels of the β-catenin mRNA and protein by directly interacting with the mRNA-stabilizing protein, HuR, thereby mediating resistance of CRC cells to oxaliplatin^210^. Oxaliplatin-resistant colon cancer cells secrete exosomal hsa_circ_0005963, miR-46146, and miR-208b molecules, which can effectively mediate the resistance of recipient cells to oxaliplatin *via* different pathways. Hsa_circ_0005963 can be transferred to sensitive CRC cells and cause oxaliplatin resistance by inhibiting glycolysis through the miR-122/PKM2 pathway^209^. Exosomal miR-46146 directly targets PDCD10 in recipient cells, contributing to the acquisition of chemotherapeutic resistance^211^. Exosomal miR-208b is delivered to recipient T cells to promote Treg expansion by targeting PDCD4, which results in tumor growth and decreased sensitivity to oxaliplatin therapy^212^.

Exosomes also contribute to 5-FU resistance in CRC patients. For example, miR-21 is significantly upregulated in exosomes derived from colon cancer cells compared to healthy human colon epithelial cells and is involved in inducing resistance to 5-FU. Compared with each monotherapy, simultaneous delivery of 5-FU and an miR-21 inhibitor oligonucleotide (miR-21i) to cancer cells *via* engineered exosomes can effectively reverse the drug resistance and significantly enhance the cytotoxicity toward 5-FU-resistant colon cancer cells, suggesting that combined delivery of functional small RNAs and anticancer drugs *via* exosomes is a potential approach to reversing drug resistance and enhancing the effectiveness of cancer treatment in clinical practice^214,215^. MiR-204-5p is another exosomal molecule that mediates the resistance of CRC patients to 5-FU^216^.

In addition, serum exosomal miR-92a-3p levels have been shown to be significantly higher in patients resistant to 5-FU/oxaliplatin than the chemotherapy-sensitive group, suggesting that exosomal miR-92a-3p may be a marker for predicting chemotherapeutic sensitivity in CRC patients^202^. Upregulated expression of hsa_circ_0032883, hsa_circ_0000338, and hsa_circ_0066629 was demonstrated in exosomes from FOLFOX-resistant HCT116 cells; hsa_circ_0000338 was absorbed by drug-sensitive cells *via* exosomes and could contribute to increasing drug resistance of recipient cells^205^.

CSCs are inherently resistant to chemotherapy, but the mechanisms are not well understood. Exosomes may partly explain this resistance of CSCs. Exosomes secreted by CAFs carry Wnt molecules and can reprogram CRC cells into CSCs, thus promoting the resistance of CRC patients to chemotherapy drugs, including oxaliplatin and 5-FU, which suggests that blocking the process of exosomal delivery of Wnt molecules may help improve drug sensitivity and therapeutic effects in CRC patients^217^. Similarly, exosomes secreted by CAFs can transfer lncRNA H19 into CRC cells, which has been demonstrated to not only promote stem cell properties, but also drug resistance of CRC cells *in vivo* and *in vitro*. The specific mechanism involves lncRNA H19 acting as a competing endogenous RNA sponge for miR-141, while miR-141 significantly inhibits the stemness of CRC cells^208^.

With low toxicity and immunogenicity, exosomes are ideal delivery vehicles for drug-loading therapy^223^. Studies have shown that exosomes containing iRGD peptides exhibit efficient targeting *in vivo*. Lin et al.^218^ loaded iRGD-modified exosomes with siCPT1A to specifically deliver CPT1A siRNA to colon cancer cells, thus reversing oxaliplatin resistance by regulating fatty acid oxidation. Moreover, iRGD exo-si showed stronger CPT1A inhibition than exo-si both *in vitro* and *in vivo*, suggesting that iRGD-modified exosomes are a better candidate for siRNA targeting colon cancer. MSCs are pluripotent stem cells with the ability of self-renewal and multi-directional differentiation, which have great clinical applications. Han and colleagues constructed iRGD-Lamp2b-modified MSCs, isolated exosomes, and loaded anti-miRNA-221 oligonucleotide (AMO) into the exosomes by electroporation. Exosomes loaded with AMO effectively inhibit the proliferation and clonal formation of colon cancer cells, thus having an anti-tumor role^219^. Moreover, packaging doxorubicin into exosomes secreted by MSCs by electroporation and covalent modification of exosome surface amine groups by carboxyl-terminal MUC1 aptamer formation *via* amide linkage can provide selective guided drug delivery for CRC treatment^220^. Li et al.^221^ conjugated a high-density A33 antibody to carboxy Fe3O4 superparamagnetic nanoparticles (A33Ab-US) and loaded doxorubicin into exosomes derived from A33-positive LIM1215 cells to form a complex (A33Ab-US-Exo/Dox). *In vivo* experiments showed that A33Ab-US-Exo/Dox had good tumor targeting, inhibited tumor growth, prolonged mouse survival, and reduced cardiac toxicity. The findings suggest that exosomes coated with high-density antibodies to target ligands may be a novel strategy for delivering doxorubicin and enhancing its therapeutic efficacy. In addition, Hosseini et al.^222^ successfully developed the doxorubicin-loaded AS1411 (anti-nucleolin) aptamer surface-functionalized exosome (DOX-Apt-Exo) to treat CRC, which showed that AS1411 aptamer-modified exosomes can be used as a safe and effective targeted drug delivery system for the clinical applications of CRC. In summary, exosomes, as loaded drug delivery vehicles, have shown amazing value in the treatment of CRC. The potential exosomal biomarkers related to CRC chemoresistance and drug loading therapy are shown in **Table 1**.

## Significance of exosomes in CRC immunotherapy

More recently, immunotherapy based on immune checkpoint inhibitors (ICIs) has improved clinical outcomes in CRC patients with MSI^224^. MSI mainly occurs in DNA mismatch repair defect-type CRC cells, which accumulate a large number of so-called small insertion/deletion mutations that are mainly concentrated in short and repetitive DNA sequences (microsatellites)^225,226^. Repeated mutation of TGFBR2 is the driving factor of MSI tumorigenesis, and exosomes reflect the MSI status and coding mononucleotide repeat frameshift allele pattern of MSI CRC cells^227^. Fricke et al.^228^ found that differences in the characteristics of exosomal proteins secreted by MSI CRC cell lines are mainly dependent on the TGFBR2 expression status of donor cells and these exosomes lead to increased levels of PDGF-B protein secreted by HepG2 receptor cells in a TGFBR2-dependent manner. In addition, Fricke and colleagues^229^ revealed abnormal expression of multiple miRNAs in TGFBR2-deficient MSI CRC cells and the secreted EVs.

**Figure 3:**
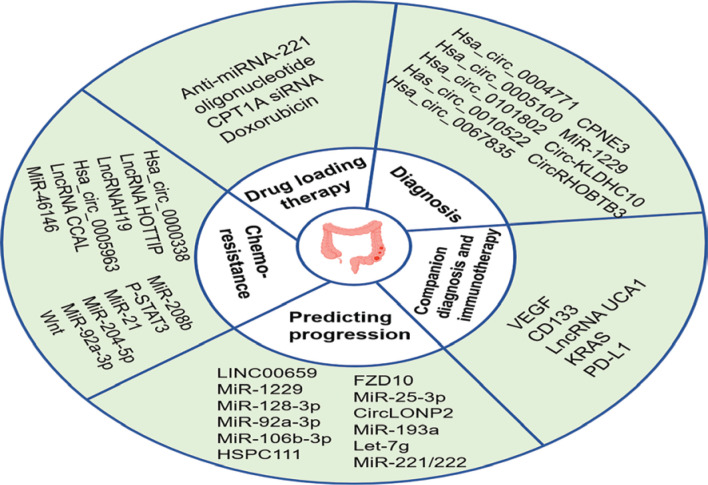
Exosome biomarkers in CRC progression, diagnosis, and treatment. Because exosomes carrying a variety of bioactive molecules reflect the characteristics of the parent cells and can be detected in multiple body fluids, exosomes have great potential as biomarkers for the diagnosis, treatment, and prediction of CRC progression. In addition, exosomes can also be used as a adjunctive diagnosis for CRC patients and a dynamic observation indicator reflecting disease changes to guide personalized medicine. With low toxicity and immunogenicity, exosomes are ideal drug delivery carriers. Numerous engineered exosomes have been designed for drug delivery therapy in CRC.

Unfortunately, < 15% of CRC patients present with MSI, and the majority of CRC patients present with MMR-proficient (pMMR) or MSS phenotypes, and ICI therapy has little clinical benefit for these patients^230–233^. Therefore, there is an urgent need to identify strategies that enhance the effectiveness of treatment for these patients. As messengers of information exchange between cells, exosomes carrying bioactive molecules have great potential and value in enhancing the effect of immunotherapy. PD-L1 antibodies can be bound to and consumed by exosomal PD-L1 in the bloodstream^130^. One study found that sulindac downregulates PD-L1 by blocking the NF-κB signaling pathway, thereby reducing the amount of exosomal PD-L1 secreted by tumor cells and enhancing the efficacy of PD-L1 immunotherapy in CRC patients with the pMMR phenotype^234^. **Figure 3** showed that exosome biomarkers in CRC progression, diagnosis, and treatment.

## Conclusions and outlook

In this review the regulatory role of exosomes in the progression of CRC and the potential clinical value of exosomes in the diagnosis, treatment, and prognosis of CRC are described. TME is a complex and dynamic environment that regulates tumor behavior, which is mainly composed of tumor cells, various immune cells, stromal cells, and other non-cellular components. As communicators of intercellular information, exosomes can mediate the crosstalk between tumor cells and other cells within the TME to remodel the TME, then regulate tumor progression. This review discussed and summarized the role of exosomes in mediating the crosstalk between CRC and other cells in TME. CRC-derived exosomes promote the differentiation of macrophages into the M2 phenotype, enhance the immunosuppressive function of MDSCs, activate the protumor phenotype of neutrophils, participate in the regulation of B cells, inhibit the activity of T cells, and participate in the activation of CAFs, thus leading to decreased immunity and CRC progression. Notably, not all CRC-derived exosomal molecules suppress immune function; some CRC-derived exosomal molecules may enhance immune function. In addition, immune cells can influence CRC cells by releasing exosomes, such as DCs and NK cells, and exosomes secreted by CAFs also have a key role in CRC progression. Taken together, exosome-mediated interactions between CRC and various other cells in TME play a crucial role in the progression of CRC. Moreover, metastasis is a key event in CRC progression and the main cause of death in CRC patients. Therefore, we also summarized the role of exosomes in CRC metastasis. Exosomes are stable message carriers for a variety of biomolecules and take part in each step of CRC progression. Mounting evidence indicates that some exosomal molecules are useful biomarkers for the early diagnosis and companion diagnosis or prediction of distant metastasis or drug resistance in CRC, and engineered exosomes can be used as delivery systems to load drugs for the treatment of CRC. Some representative exosomal molecules are discussed in this review. Although great progress has been made, further studies are needed to elucidate the roles of exosomes as clinical biomarkers for CRC and their roles in the crosstalk between CRC and immune cells.

Exosomes have shown promising potential in the treatment of CRC, but there are still many limitations and challenges to overcome before exosomes can be successfully applied as biomarkers in the clinic. The main obstacles that limit the widespread clinical use of exosome biomarkers are their heterogeneity, the presence of non-vesicular extracellular substances, such as exomeres^235^ and supermeres^236^, and the lack of standardized and reliable methods for mass production of exosomes. Understanding exosome heterogeneity and the composition and functional properties of non-vesicular extracellular nanoparticles is crucial for identifying biomarkers and designing drug treatment strategies. Secondly, it is important to improve the efficiency of intercellular delivery of exosomes and ensure the specific delivery of exosome components. Studies have found that some proteins and nucleic acids will degrade or form insoluble nucleic acid aggregates during the process of loading into exosomes, which will affect the delivery efficiency of exosomes. In addition, the mechanisms by which different cell types influence the delivery properties of exosomes are largely unknown, and exosomes derived from the same cell can also show differences in properties, so exosomes need to be properly characterized before application in clinical therapy. The complexity of TME changes dynamically, as well as the fact that exosomes contain many currently unknown components. Targeting one exosomal component for treatment may induce a compensatory response of the TME or compensatory changes in other components of the exosome. Finally, due to the differences between animal models and human experiments, the dose of exosomes cannot be uniformly regulated. At present, there are few clinical studies with a focus on the diagnosis and treatment of CRC based on exosomes. Therefore, we need to conduct additional studies to standardize the effective conversion of data between animal experiments and clinical trials to promote the clinical application of exosomes.
